# The Fecal Microbiome and Metabolome of Pitt Hopkins Syndrome, a Severe Autism Spectrum Disorder

**DOI:** 10.1128/mSystems.01006-21

**Published:** 2021-11-30

**Authors:** Amanda H. Dilmore, Daniel McDonald, Tanya T. Nguyen, James B. Adams, Rosa Krajmalnik-Brown, Emmanuel Elijah, Pieter C. Dorrestein, Rob Knight

**Affiliations:** a Department of Pediatrics, School of Medicine, University of California, San Diego, San Diego, California, USA; b Department of Psychiatry, UC San Diego, San Diego, California, USA; c Sam and Rose Stein Institute for Research on Aging, UC San Diego, San Diego, California, USA; d School for Engineering of Matter, Transport and Energy, Arizona State University, Tempe, Arizona, USA; e Biodesign Center for Health through Microbiomes, Arizona State University, Tempe, Arizona, USA; f School of Sustainable Engineering and the Built Environment, Arizona State University, Tempe, Arizona, USA; g Pharmaceutical Sciences, University of California, San Diego, San Diego, California, USA; h Collaborative Mass Spectrometry Innovation Center, Skaggs School of Pharmacy, University of California, San Diego, San Diego, California, USA; i Center for Microbiome Innovation, Jacobs School of Engineering, University of California, San Diego, San Diego, California, USA; j Department of Computer Science, Jacobs School of Engineering, University of California, San Diego, San Diego, California, USA; k Department of Bioengineering, University of California, San Diego, San Diego, California, USA; University of California, San Francisco

**Keywords:** autism spectrum disorders, computational biology, microbiome

## Abstract

Alterations to the gut microbiome have been reported between children with autism spectrum disorders (ASDs) and typically developing (TD) children. Characterizing these differences has led to the proposal of new treatments for ASD, such as probiotic interventions and fecal matter transplants. However, no study to date has characterized the gut microbiome or metabolome in Pitt Hopkins syndrome (PTHS), a severe ASD with a high incidence of gastrointestinal (GI) disturbances such as constipation. Here, we surveyed the gut microbiome and metabolome in a cohort of PTHS individuals and their unaffected parents. We focused our analysis on Clostridium bolteae, a microbe previously associated with ASD known to chemically modify bile acids in the gut. PTHS individuals carry a higher load of C. bolteae than their parents as well as both ASD and non-ASD individuals from the American Gut Project cohort. Specific metabolites were associated with PTHS, including bile acids and sphingosines. With a metadata reanalysis tool, we found that PTHS-associated metabolites have previously been identified in inflammatory bowel disease and obesity patients. These results suggest microbial involvement in PTHS, but further research must be performed to clarify the exact mechanisms through which microbes may act. Furthermore, new associations between PTHS-specific metabolites and other conditions may lead to additional therapeutic options for PTHS individuals.

**IMPORTANCE** GI disturbances in ASD such as severe constipation can be medically significant and often require medication. This is especially true for individuals with PTHS, suggesting that the gut microbiome may be involved in PTHS’s pathology. Revealing associations between specific gut microbes and PTHS may allow the development of new therapeutics or the application of existing therapeutics to ease day-to-day challenges encountered by PTHS individuals. In this study, we characterized an association between *C. bolteae* and PTHS, in addition to metabolites linked to both PTHS and *C. bolteae*. We also identified other microbiome-involved medical conditions where PTHS-associated metabolites have been isolated. Utilizing common metabolites to identify conditions with similar phenotypes may suggest new therapeutic options for GI-related symptoms.

## OBSERVATION

Autism spectrum disorders (ASDs) are a heterogeneous group of neurodevelopmental disorders characterized by deficits in communication and social interaction and repetitive stereotyped behaviors (1). No single etiology of ASD has been identified, and current hypotheses suggest both genetic and environmental contributions (2, 3). On the environmental side, alterations in the gut microbiome are reported in individuals with ASD compared to unaffected children (4). Furthermore, both gnotobiotic animal and probiotic studies suggest a potential causative role for the gut microbiome in behavioral and neuropathological endophenotypes in human ASD (5–8).

Pitt Hopkins syndrome (PTHS) (9, 10) is a rare and extreme form of ASD that is caused by a pathogenic variant of the *TCF4* gene found on chromosome 18q21.2 (11–13). PTHS is characterized by severe intellectual disability and psychomotor delay, facial dysmorphism, hyperventilation-apneic spells, stereotypic movements, and seizures. Gastrointestinal (GI) disturbance, especially severe constipation, is the most common extraneurological manifestation of PTHS; it can cause significant pain and often requires medication. The pathology of GI abnormalities in ASD appears to be at least partially related to the gut microbiome. Despite substantial evidence of its alterations and potential therapeutic value in ASD, no study to our knowledge has examined the composition of the fecal microbiome or its resultant metabolites in PTHS. Thus, the purpose of this study was to characterize the gut microbiome and metabolome of 39 children with PTHS compared to 46 unaffected family members to understand how each contributes to clinical pathology (see Table 1 for demographic and clinical information). This characterization is critical for targeting interventions to improve the quality of life of these individuals.

**TABLE 1:** Demographic and clinical characterization of the 39 Pitt Hopkins syndrome patients and their 46 unaffected family members^*a*^

Metric	Value for group	
	Pitt Hopkins syndrome patients (*n* = 39)	Unaffected family members (*n* = 46)
Mean age (yrs) ± SD (no. of individuals)	10.18 ± 8.50 (39)	39.42 ± 9.78 (38)
% (no.) of females	60.5 (38)	60.5 (43)
% (no.) of Caucasian individuals	87.2 (39)	90.0 (40)
% (no.) of individuals with USA as country of residence	92.3 (39)	90.9 (44)
% (no.) of individuals with antibiotic use in the last yr	51.3 (39)	38.5 (39)
% (no.) of individuals with at least weekly use of probiotic	48.6 (37)	17.1 (41)
% (no.) of individuals with normal bowel movement quality	44.7 (38)	90.0 (40)
% (no.) of individuals in overweight or obese BMI category	8.57 (35)	56.1 (41)

a Notably, a large share of PTHS patients have used antibiotics within the last year and take probiotics regularly compared to their family members. Furthermore, PTHS patients are less likely to be overweight or obese and are more likely to have abnormal stool quality. BMI, body mass index.

Our analysis narrows in on Clostridium bolteae, a microbe that is significantly more abundant in ASD children’s stool and is associated with abdominal infections (14, 15). C. bolteae’s ability to conjugate bile acids in the gut has been thoroughly characterized (16). Since dysregulated bile acid metabolism has been associated with GI dysfunction in ASD model mice and GI problems are a hallmark of PTHS, we were interested in whether *C. bolteae* and bile acids would be more common in the gut of PTHS individuals (17).

### Metagenomics and 16S rRNA gene amplicon results.

**FIG 1:**
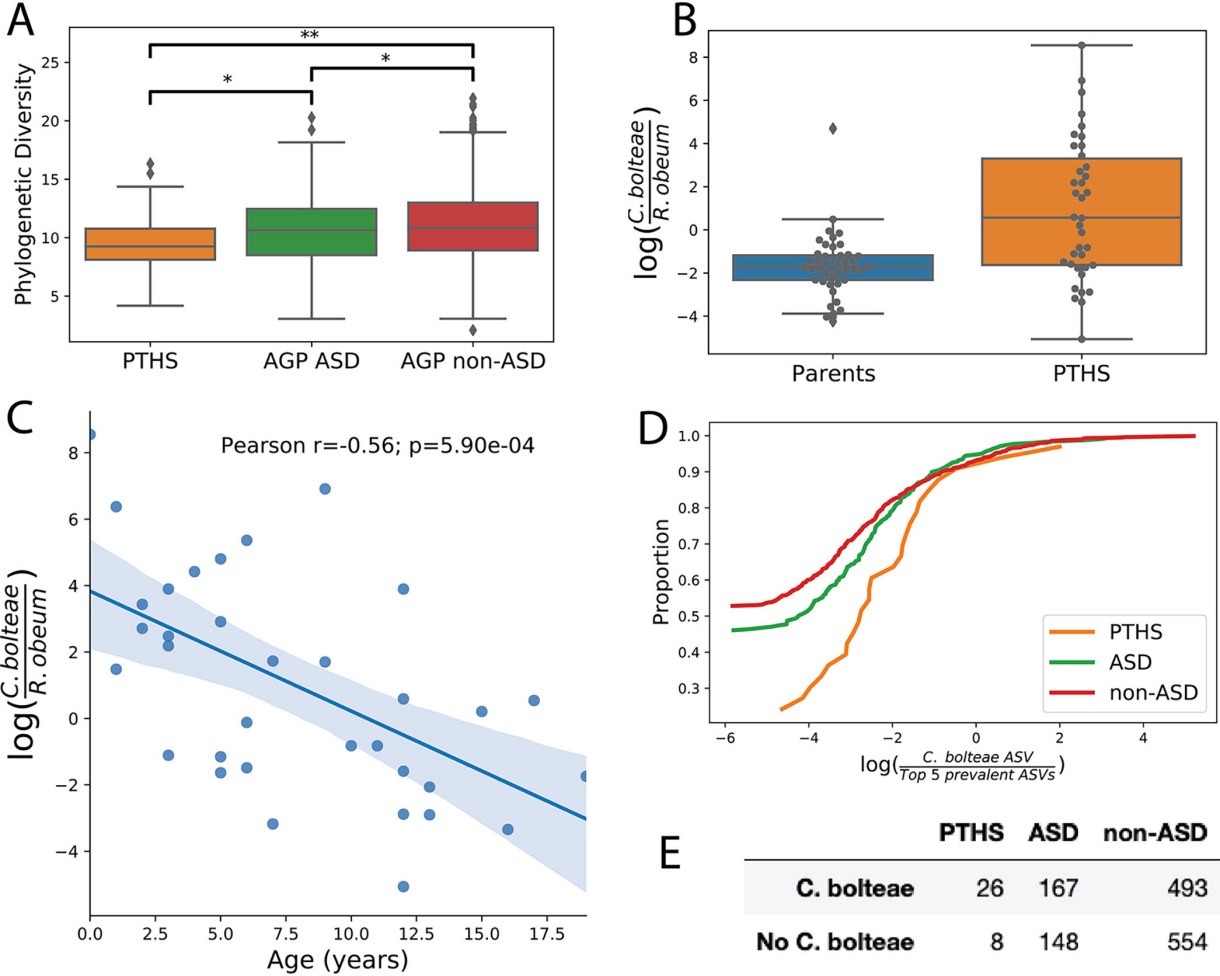
Clostridium bolteae associations in PTHS, examined due to its previous observations with ASD. (A) Using 16S rRNA V4 sequencing data, we compared PTHS individuals to individuals with a self-reported diagnosis of ASD and those without a diagnosis of ASD from the American Gut Project. All individuals selected were under 20 years old, and only samples within 1 year from a PTHS-affected individual were retained. Consistent with previous literature, we observed lower alpha diversity in both PTHS and ASD individuals (PTHS versus AGP ASD, U = 3,668 and *P* = 0.013; PTHS versus AGP non-ASD, U = 11,372 and *P* = 0.001; AGP ASD versus AGP non-ASD, U = 124,957 and *P* = 0.039 [by a Mann U test]). *, *P* < 0.05; **, *P* < 0.005. (B) Using shallow shotgun sequencing data, the log ratio of *C. bolteae* to *R. obeum* (picked as highly prevalent) was elevated in PTHS-affected individuals compared to their parents (*t* = 4.015; *P* = 1.49e−4 [by a *t* test]). (C) Due to the age differences in the groups, we examined *C. bolteae* specifically in PTHS-affected individuals under 20 years old, observing a decreasing ratio associated with age. (D and E) A single 16S amplicon sequence variant (ASV) was found to have an exact match to one of the two *C. bolteae* genomes observed in the shotgun assessment. This ASV was observed in both ASD-positive and -negative individuals within the AGP. (D) Empirical cumulative distribution functions of the *C. bolteae* ASV relative to the top five prevalent ASVs. (E) *C. bolteae* presence/absence contingency table. The *C. bolteae* ASV presence in PTHS is significant by Fisher’s exact test (PTHS versus ASD, odds ratio = 2.88 and *P* = 0.01; PTHS versus non-ASD, odds ratio = 3.65 and *P* = 7.6e−4).

It has previously been reported that gut microbiome alpha diversity is lower in ASD than in typically developing (TD) individuals (18), so we extended this comparison to include PTHS. Specifically, we measured Faith’s phylogenetic diversity among PTHS, ASD, and non-ASD individuals by combining our 16S rRNA V4 sequencing data with American Gut Project (AGP) data (Fig. 1A). We restricted the analysis to individuals under the age of 20 years to mitigate age-related changes in gut microbial diversity, as most PTHS individuals surveyed are young. Consistent with previous observations, we observed lower alpha diversity in both PTHS and ASD individuals (PTHS versus AGP ASD, U = 3,668 and *P* = 0.013; PTHS versus AGP non-ASD, U = 11,372 and *P* = 0.001; AGP ASD versus AGP non-ASD, U = 124,957 and *P* = 0.039 [by a Mann U test]). We assessed differences in beta diversity among PTHS, ASD, and non-ASD (from the AGP) individuals using permutational multivariate analysis of variance (PERMANOVA), observing significant PTHS-versus-ASD (pseudo-*F* = 4.823; *P* = 0.003) and PTHS-versus-non-ASD (pseudo-*F* = 4.086; *P* = 0.007) differences in weighted UniFrac distances (see Table S2 in the supplemental material). We then narrowed our focus to *C. bolteae*, a microbe with known associations with ASD (14, 15, 18). Shotgun metagenomics found *C. bolteae* to be elevated relative to the highly prevalent reference organism Ruminococcus obeum in PTHS individuals compared to their unaffected parents (Fig. 1B) (*t* = 4.015; *P* = 1.49e−4 [by a *t* test]). Furthermore, within affected individuals younger than 20 years of age, we observed a negative correlation between the log ratio of *C. bolteae* to R. obeum and age (Fig. 1C) (Pearson *r* = −0.56; *P* = 5.90e−4). To disentangle whether this correlation is an artifact of age, we compared the relative abundance of *C. bolteae* in PTHS individuals under 20 years of age (*n* = 34) to those in individuals with a self-reported medical diagnosis of ASD (*n* = 315) and non-ASD individuals also younger than 20 years of age (*n* = 1,047) from the AGP cohort. A single 16S amplicon sequence variant (ASV) had an exact match to one of the *C. bolteae* genomes observed in the shotgun assessment. The 16S data indicate that a significantly larger proportion of PTHS individuals have high relative abundances of *C. bolteae* than either ASD or non-ASD individuals (Fig. 1D and E) (PTHS versus ASD, odds ratio = 2.69 and *P* = 0.017; PTHS versus non-ASD, odds ratio = 3.65 and *P* = 7.62e−4 [by Fisher’s exact test]). This suggests that the higher relative abundance of *C. bolteae* in PTHS individuals than in their parents is more related to their PTHS status than to the differences in the ages of the cohorts. We further examined the relationship between *C. bolteae* and age in the 16S data. For a compositional additive log ratio (ALR) transform, we used the *C. bolteae* ASV with 100% sequence identity to the genome record from the shotgun data and the top 5 most prevalent 16S ASVs and correlated this log ratio with the age of the individual. PTHS and ASD were nonsignificant, whereas the non-ASD individuals exhibited a negative correlation (Pearson *r* = −0.07; *P* = 0.0160) (Fig. S1).

10.1128/mSystems.01006-21.2 FIG S1 Relationship between *C. bolteae* and age in the 16S data. There is no significant correlation between the abundance of the *C. bolteae* ASV and age in the PTHS (Pearson *r* = −0.07; *P* = 0.700) (A) or ASD (Pearson *r* = −0.05; *P*  = 0.379) (B) data, but there is a significant negative correlation between the *C. bolteae* ASV and age in non-ASD individuals (Pearson *r* = −0.07; *P* = 0.0160) (C). Download FIG S1, TIF file, 0.2 MB. Copyright © 2021 Dilmore et al. 2021 Dilmore et al. https://creativecommons.org/licenses/by/4.0/ This content is distributed under the terms of the Creative Commons Attribution 4.0 International license.

10.1128/mSystems.01006-21.4 TABLE S2 Beta diversity analysis of metagenomic data. We performed the following beta diversity analyses: unweighted UniFrac, weighted UniFrac, and robust Aitchison (C. Martino, J. T. Morton, C. A. Marotz, L. R. Thompson, et al., mSystems 4:e00016-19, 2019, https://doi.org/10.1128/mSystems.00016-19; C. Lozupone and R. Knight, Appl Environ Microbiol 71:8228–8235, 2005, https://doi.org/10.1128/AEM.71.12.8228-8235.2005). We then used PERMANOVA to test for between-group differences in beta diversity. We found significant differences in unweighted and weighted UniFrac distances between PTHS and ASD individuals and significant differences in unweighted UniFrac, weighted UniFrac, and robust Aitchison values between PTHS and non-ASD individuals. Download Table S2, PDF file, 0.03 MB. Copyright © 2021 Dilmore et al. 2021 Dilmore et al. https://creativecommons.org/licenses/by/4.0/ This content is distributed under the terms of the Creative Commons Attribution 4.0 International license.

### Metabolomics results.

**FIG 2:**
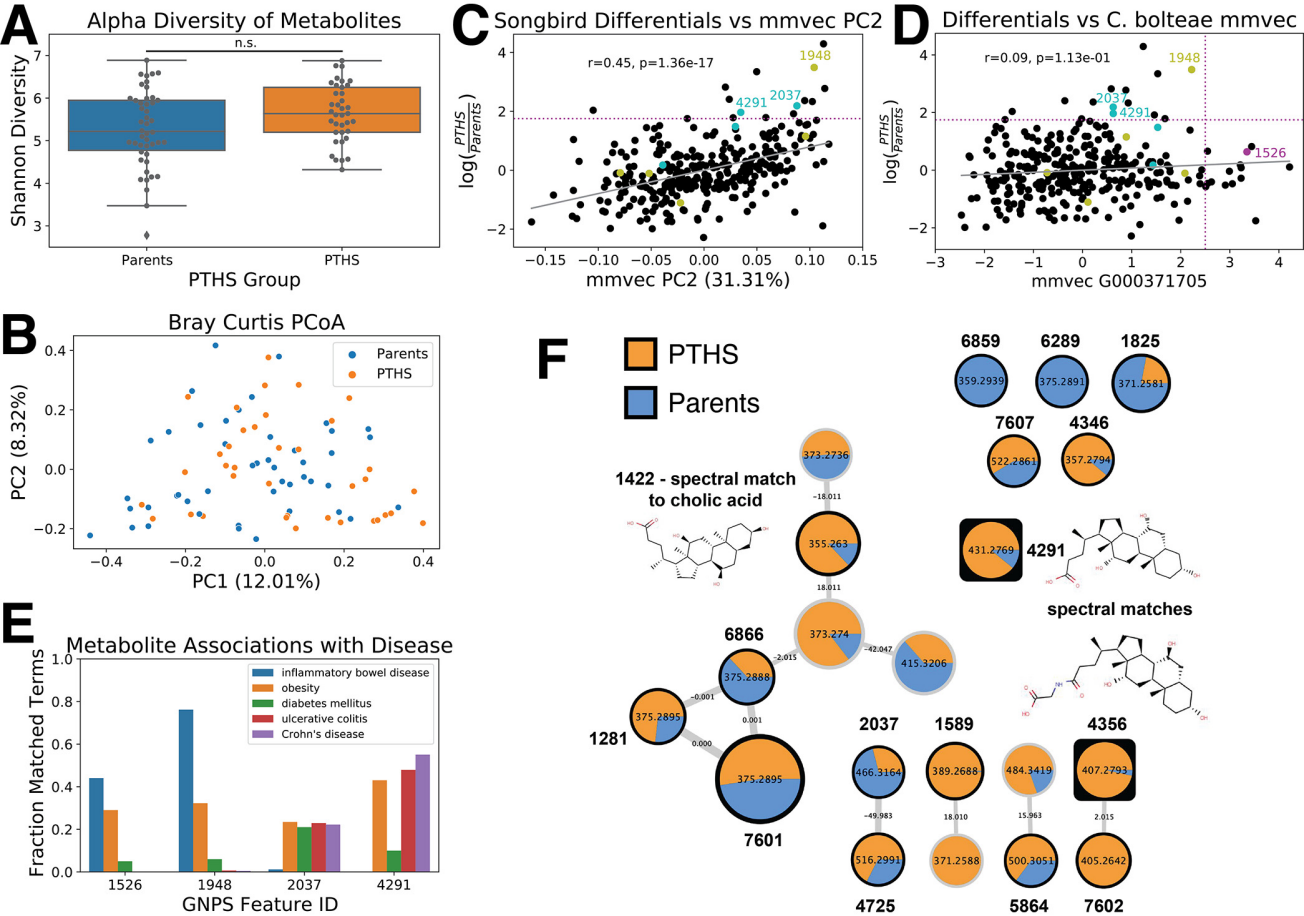
Multi-omic associations with PTHS. (A) We observed no significant difference in the alpha diversity of metabolites between PTHS individuals and their parents (*H* = 3.324; *P* = 0.0683 [by a Kruskal-Wallis test]). n.s., not significant. (B) Principal-coordinate analysis (PCoA) representation of the Bray-Curtis dissimilarity indices for PTHS individuals and their parents. While parents and PTHS individuals do not cluster distinctly, there is a significant difference in their Bray-Curtis dissimilarities (pseudo-*F* statistic = 2.06076; *P* = 0.004 [by PERMANOVA]). (C) The top metabolites associated with PTHS were identified by Songbird trained over the formula “*y* ∼ pths_status.” These Songbird differentials correlate with mmvec PC2 (Pearson *r* = 0.45; *P* = 1.37e−17). PTHS-enriched features were selected as those with log ratios of >1.75. Level 2- or 3-annotated compounds are indicated by labeling with their Global Natural Product Social Molecular Networking (GNPS) feature identifiers. Cyan indicates that the feature is a bile acid, while yellow indicates that it is a phosphocholine. (D) The top features associated with PTHS by Songbird plotted against their association with *C. bolteae* by mmvec (Pearson *r* = 0.09; *P* = 0.113). Features highly associated with *C. bolteae* were selected as those with log conditional probabilities of >2.5. Again, level 2- or 3-annotated compounds are indicated by labeling with their GNPS feature identifiers. Cyan indicates that the feature is a bile acid, yellow indicates that it is a phosphocholine, and magenta indicates that it is a bile acid precursor. (E) The top annotated features identified from Songbird and mmvec analyses were examined with ReDU’s Chemical Explorer for their associations with disease. These features appear frequently in IBD, obesity, diabetes, ulcerative colitis, and Crohn’s disease data sets deposited in GNPS. (F) Molecular network of bile acids identified by feature-based molecular networking (FBMN). The pie charts represent the proportions of the summed ion intensities for each feature observed in PTHS individuals and their parents. A black outline indicates that the node has an associated level 2 or 3 annotation as defined by the Metabolomics Standard Initiative (22), while a square outline indicates that the node is a top feature by Songbird.

Unlike in the metagenomic results, we observed no significant differences in metabolite alpha diversity between PTHS individuals and their parents (Fig. 2A) (Kruskal-Wallis statistic = 3.324; *P* = 0.0683). However, we observed a significant difference in metabolite beta diversity (Fig. 2B) (PERMANOVA statistic = 2.06076; *P* = 0.004). To identify metabolites associated with PTHS, we performed a differential analysis with the Songbird multinomial model (19); we found that these differentials correlated with mmvec (20) PC2 (Pearson *r* = 0.45; *P* = 1.37e−17) (Fig. 2C; see also Text S1 in the supplemental material for more background on Songbird and mmvec). Furthermore, given the association between *C. bolteae* and PTHS, we employed mmvec to identify metabolites associated with *C. bolteae*. No association was found between metabolite Songbird differentials and their mmvec conditional probabilities for *C. bolteae* genomic operational taxonomic unit (gOTU) G000371705 (Fig. 2D) (Pearson *r* = 0.09; *P* = 0.113), but those metabolites with high conditional probabilities were analyzed further (Table S1). The top Songbird and mmvec metabolites were examined with a metadata reanalysis tool (21), which showed that they are often isolated from patients with irritable bowel disease (IBD), obesity, and Crohn’s disease, among other conditions (Fig. 2E). Finally, as we are interested in the association between *C. bolteae* and bile acids, we examined all level 3-annotated bile acids by feature-based molecular networking (22, 23) (Fig. 2F and Table 2). Many bile acids appear to be enriched in PTHS patients relative to their parents, particularly those that are chemically modified, suggesting microbial involvement in PTHS.

**TABLE 2:** Bile acid spectral matches^*a*^

Feature ID	*m/z*	RT^*b*^	Annotation
1281	375.2895	4.31	(R)-4-((5R,8R,9S,10S,13R,14S,17R)-10,13-Dimethyl-3-oxohexadecahydro-1H-cyclopenta[a]phenanthren-17-yl)pentanoic acid
1422	355.2630	3.56	Cholic acid
1589	389.2688	3.32	(4R)-4-((3R,5R,6S,7R,9S,10R,12S,13R,14S,17R)-3,6,7,12-Tetrahydroxy-10,13-dimethylhexadecahydro-1H-cyclopenta[a]phenanthren-17-yl)pentanoic acid
1825	371.2581	4.05	(R)-4-((3S,5S,7R,8R,9S,10S,13R,14S,17R)-3,7-Dihydroxy-10,13-dimethyl-12-oxohexadecahydro-1H-cyclopenta[a]phenanthren-17-yl)pentanoic acid
2037	407.2793	3.30	(R)-4-((1R,3S,5S,7R,8S,9S,10S,12S,13R,14S,17R)-1,3,7,12-tetrahydroxy-10,13-dimethylhexadecahydro-1H-cyclopenta[a]phenanthren-17-yl)pentanoic acid
4291	431.2769	3.57	(R)-4-((3R,5S,7R,8R,9S,10S,12S,13R,14S,17R)-3,7,12-Trihydroxy-10,13-dimethylhexadecahydro-1H-cyclopenta[a]phenanthren-17-yl)pentanoic acid
4346	357.2794	7.04	(4R)-4-((5S,7S,9S,10S,12R,13R,14S,17R)-7,12-dihydroxy-10,13-dimethylhexadecahydro-1H-cyclopenta[a]phenanthren-17-yl)pentanoic acid
4356	466.3164	3.25	((R)-4-((3R,5S,7S,8R,9S,10S,12S,13R,14S,17R)-3,7,12-Trihydroxy-10,13-dimethylhexadecahydro-1H-cyclopenta[a]phenanthren-17-yl)pentanoyl)glycine
4725	405.2642	3.17	(R)-4-((3R,5S,8R,9S,10S,13R,14S,17R)-3-Hydroxy-10,13-dimethyl-7,12-dioxohexadecahydro-1H-cyclopenta[a]phenanthren-17-yl)pentanoic acid
5864	500.3051	4.03	2-((4R)-4-((3R,5S,7R,9S,10S,13R,14S,17R)-3,7-Dihydroxy-10,13-dimethylhexadecahydro-1H-cyclopenta[a]phenanthren-17-yl)pentanamido)ethane-1-sulfonic acid
6289	375.2891	5.46	(R)-4-((5R,8R,9S,10S,13R,14S,17R)-10,13-Dimethyl-3-oxohexadecahydro-1H-cyclopenta[a]phenanthren-17-yl)pentanoic acid
6859	359.2939	5.15	(R)-4-((3S,5R,8R,9S,10S,13R,14S,17R)-3-Hydroxy-10,13-dimethylhexadecahydro-1H-cyclopenta[a]phenanthren-17-yl)pentanoic acid
6866	375.2888	3.94	(R)-4-((5R,8R,9S,10S,13R,14S,17R)-10,13-Dimethyl-3-oxohexadecahydro-1H-cyclopenta[a]phenanthren-17-yl)pentanoic acid
7601	375.2895	4.24	(R)-4-((5R,8R,9S,10S,13R,14S,17R)-10,13-Dimethyl-3-oxohexadecahydro-1H-cyclopenta[a]phenanthren-17-yl)pentanoic acid
7602	516.2991	3.49	2-((R)-4-((3R,5S,7S,8R,9S,10S,12S,13R,14S,17R)-3,7,12-Trihydroxy-10,13-dimethylhexadecahydro-1H-cyclopenta[a]phenanthren-17-yl)pentanamido)ethane-1-sulfonic acid
7607	522.2861	4.03	2-((R)-4-((3S,5S,7S,8R,9S,10S,13R,14S,17R)-3,7-Dihydroxy-10,13-dimethylhexadecahydro-1H-cyclopenta[a]phenanthren-17-yl)pentanamido)ethane-1-sulfonic acid

a Feature information for all compounds annotated as bile acids is shown. The feature identifiers outlined here match up to the compounds in the molecular network in Fig. 2F. The annotations are all level 3, as defined by the Metabolomics Standards Initiative (19).

b RT, retention time (seconds).

10.1128/mSystems.01006-21.1 TEXT S1 Supplemental methods, additional information about the bioinformatic tools Songbird and mmvec, and references specific to the supplemental material. Download Text S1, PDF file, 0.2 MB. Copyright © 2021 Dilmore et al. 2021 Dilmore et al. https://creativecommons.org/licenses/by/4.0/ This content is distributed under the terms of the Creative Commons Attribution 4.0 International license.

10.1128/mSystems.01006-21.3 TABLE S1 Top metabolite features identified from mmvec and Songbird. The top 15 features from mmvec (association with *C. bolteae* gOTU G000371705 reported here) and Songbird [formula, *y* ∼ pths_status; log(PTHS/Parents)] are detailed here. The feature ID refers to the identification given in the GNPS feature-based molecular networking job. The annotations given are either level 2 or level 3, as defined by the Metabolomics Standards Initiative (22). Download Table S1, PDF file, 0.1 MB. Copyright © 2021 Dilmore et al. 2021 Dilmore et al. https://creativecommons.org/licenses/by/4.0/ This content is distributed under the terms of the Creative Commons Attribution 4.0 International license.

### Discussion.

Multi-omic microbiome assessments are increasingly common thanks to the prospect of a more detailed understanding of the role of microbes and molecules in an environment. Here, we utilized the strength of each data layer: 16S for comparison against a large publicly available data set, shallow shotgun sequencing for the identification of species-level features, and metabolomics for the characterization of molecular features. These data layers were then used in combination: resolving a putative ASV linked to *C. bolteae* allowed us to compare it to public 16S data, and an integrative method gave us the ability to predict which microbes and molecules are probabilistically related.

The combination of these -omic strategies suggests an increased burden of *C. bolteae* within individuals with PTHS and provides evidence of a unique molecular repertoire with microbial relationships. Chronic gastrointestinal issues are a hallmark of PTHS, and many individuals suffer from frequent seizures anecdotally related to the extent of constipation. *C. bolteae*’s modification of bile acids has experimentally been shown in murine models (16), and while the same conjugated bile acids were not observed in this study, associations between PTHS and bile acids and between *C. bolteae* and bile acid precursors were observed. Alterations to bile acid metabolism have previously been linked to constipation (24), and in canines, bile acid tests are recommended if seizures are observed as a proxy for liver function (25).

These results are not mechanistic, and further work is necessary to determine the exact relationships between bile acids and *C. bolteae*. Specifically, our bile acid conclusions are limited by our lack of standard-matched level 1 annotations (22). We are able to detect several bile acids with level 3 annotations, but future studies should look to characterize individual bile acids in PTHS with specific standards. Furthermore, as Pitt Hopkins syndrome is a rare disease, the overall sample size considered in this study is limited, with reduced statistical power. Based on -omics observations, we advocate for assessments of microbial involvement in TCF4 mutation murine models and whether alterations to the microbiome (e.g., humanizing and antibiotics, etc.) mitigate PTHS-like GI disturbances (26) and behaviors (27).
