# Supplementary figures and images for: The Fecal Microbiome and Metabolome of Pitt Hopkins Syndrome, a Severe Autism Spectrum Disorder
Source: mSystems. 2021 Nov 30;6(6):e01006-21. doi: 10.1128/mSystems.01006-21 (PMC8631314; doi:10.1128/mSystems.01006-21)

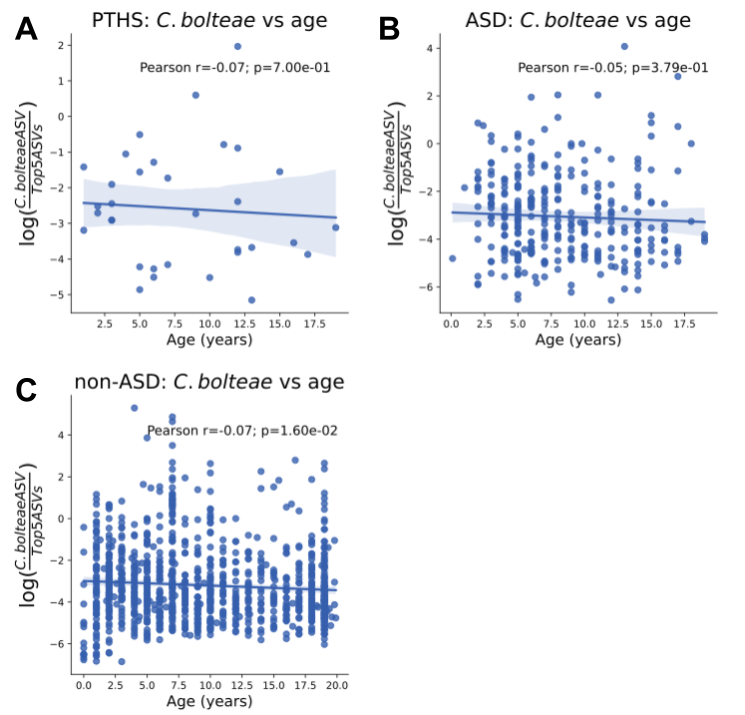

Supplement: FIG S1 [file msystems.01006-21-sf001.tif]
