# Supplementary material for: The Fecal Microbiome and Metabolome of Pitt Hopkins Syndrome, a Severe Autism Spectrum Disorder
Source: mSystems. 2021 Nov 30;6(6):e01006-21. doi: 10.1128/mSystems.01006-21 (PMC8631314; doi:10.1128/mSystems.01006-21)
Supplement: TABLE S2 [file msystems.01006-21-st002.pdf]

	**PTHS, ASD, non-ASD**	**PTHS, ASD only**	**PTHS, non-ASD only**
**Unweighted UniFrac**	pseudo-F=4.285<br>p=0.001	pseudo-F=2.881<br>p=0.001	pseudo-F=3.985<br>p=0.001
**Weighted UniFrac**	pseudo-F=3.525<br>p=0.002	pseudo-F=4.823<br>p=0.003	pseudo-F=4.086<br>p=0.007
**Robust Aitchison (DEICODE)**	pseudo-F=3.886<br>p=0.004	pseudo-F=1.069<br>p=0.341	pseudo-F=2.990<br>p=0.038
