# Supplementary material for: The Fecal Microbiome and Metabolome of Pitt Hopkins Syndrome, a Severe Autism Spectrum Disorder
Source: mSystems. 2021 Nov 30;6(6):e01006-21. doi: 10.1128/mSystems.01006-21 (PMC8631314; doi:10.1128/mSystems.01006-21)
Supplement: TEXT S1 [file msystems.01006-21-t0001.pdf]

Supplemental Methods:

(i) Participant recruitment and sample collection: At the Pitt Hopkins Research Symposium in June 2018, attending and interested families were provided American Gut Project microbiome collection kits (28) and the opportunity to voluntarily participate in the project under UC San Diego IRB protocol 141853. Diagnosis was self-reported, and no inclusion/exclusion criteria were used. Fecal Occult Blood Test cards were provided as the collection devices to stabilize nucleic acids in preparation for shotgun sequencing, and to mitigate known microbial blooms with traditional American Gut collection devices (29). Families were provided multiple collection devices to allow multiple family members to participate if they choose. A total of 108 collection devices were received.

(ii) 16S Sequencing: The V4 region of the 16S rRNA gene was amplified with barcoded primers and sequenced on the Illumina MiSeq platform, as previously described in (30), using the 515f/806rB primer pair with the barcode on the forward read (31).

(iii) Metagenomic sequencing: Shotgun sequencing libraries from a total of 5 ng (or 3.5 µl maximum) gDNA were used in a 1:10 miniaturized Kapa HyperPlus protocol with a 15-cycle PCR amplification. Libraries were quantified with the PicoGreen dsDNA assay kit, and 50 ng (or 1 µl maximum) of each library was pooled. The pool was size selected for 300 to 700 bp and sequenced as a paired-end 150-cycle run on an Illumina HiSeq 4000 sequencer at the UCSD IGM Genomics Center.

(iv) 16S & Metagenomic Analyses: Human reads from the shotgun sequencing data were removed using minimap2 2.17 (30). Adapters and quality filtering and trimming were performed using fastp 20.1 (33) in Qiita (34). Reads were recruited to the Web of Life database (35) using Bowtie2 v2.3.0 (36) using the parameters from the SHOGUN pipeline (37) and processed into Genomic Operational Units in Woltka (38).

Two genomes in the Web of Life are annotated as *Clostridium bolteae*, G000371705 and G000431175. An additive log ratio (39). transformation was performed using the *C. bolteae*

genomes as the numerator, and the genome G000436995 as the denominator; G000436995 was observed to be present in all PTHS and non-PTHS samples and picked as being fully prevalent. Mann Whitney U tests were performed using the stats module of SciPy 1.5.2 (40). Plotting was performed using Seaborn 0.10.1 (41) and matplotlib 3.2.2 (42).

16S sequencing data were processed in Qiita (34) using Deblur v1.1.0 (43) retaining the positive filtered feature table. Existing American Gut (28) 16S fecal samples associated without ASD were selected from redbiom v0.3.5 (44) using the query “`where (qiita_study_id=='10317' and env_package=='human-gut') and (age_years < 20 and asd == 'I do not have this condition')"`”, and samples from individuals with ASD using the query “`where (qiita_study_id=='10317' and env_package=='human-gut') and (age_years < 20 and asd == 'Diagnosed by a medical professional (doctor, physician assistant)')`”. Feature tables were obtained from the “Deblur-Illumina-16S-V4-150nt-780653” redbiom context. These feature tables were joined with the PTHS 16S affected individuals data using the Table.concat method of the BIOM Table API (45). Blooms previously observed in the American Gut Project were removed from all samples (29). The data were then rarefied to 1000. Unique features were inserted into Greengenes 13_8 (46, 47) using SEPP (48), followed by calculation of Faith’s Phylogenetic Diversity (PD) (49) with the q2-diversity plugin from QIIME 2 2020.8 (50). AGP ASD and non-ASD distributions of PD samples were constructed by filtering to all samples which were +/- 1 year of a PTHS affected sample; in the case of PTHS samples with similar ages, the corresponding AGP samples were only included once. Mann Whitney U tests were performed on the resulting PD distributions using the stats module of SciPy.

To determine putative *C. bolteae* amplicon sequence variants (ASV), each ASV was searched for an exact match against the *C. bolteae* genome contigs from the Web of Life. Two hits were observed, G000371705 and G000431175. The latter was removed from subsequent

consideration as it contains the forward primer, suggesting a sequencing artifact, and represents a shifted version of the other ASV. A sample in the American Gut ASD/non-ASD data or the PTHS affected individuals data was considered to contain the *C. bolteae* ASV if that feature was present in the non-rarefied feature table. Fisher's exact test was computed using SciPy (40). An additive log ratio of the ASV and the top five most prevalent ASVs in the data was constructed, and an empirical cumulative distribution function plot generated using matplotlib (42).

Summaries and plotting were performed using a Jupyter Notebook 6.1.3 with Pandas 0.25.3.

(v) Metabolomics sample preparation and data acquisition: Untargeted metabolomics was performed on fecal samples as previously described (53). Briefly, 50% methanol spiked with 2 μM sulfamethazine was added to each fecal sample (approximately 50-100 mg feces) at a volume ratio of 10 μL extraction solvent to 1 mg sample. Samples were homogenized and centrifuged, then 400 μL of the resulting supernatant were transferred to a 96-well deep-well plate, dried via a centrifugal low-pressure system (SpeedVac Plus, Savant), and stored at −80°C. Next, samples were resuspended, sonicated, and centrifuged. 100 μL of supernatant were transferred to a new shallow-well 96-well plate and diluted 20-fold. These samples were analyzed using ultra-high performance liquid chromatography (Ultimate 3000, Thermo) coupled to a quadrupole time-of-flight mass spectrometer (maXis Impact, Bruker). Chromatographic separation was accomplished using a Kinetex C18 1.7 uM, 100 Å, 2.1 mm by 50 mm column (Phenomenex) maintained at 40°C during separation. 5 uL of extract was injected per sample.

Mobile phase composition was A, LC-MS grade water with 0.1% formic acid (v/v), and B, LC-MS grade acetonitrile with 0.1% formic acid (v/v). The chromatographic elution gradient parameters were set as previously described (53). The acquired qTOF files (.d) were exported using DataAnalysis (Bruker) as .mzXML files after lock mass correction and uploaded to MassIVE (54).

(vi) LC-MS/MS data processing: .mzXML files were downloaded from MassIVE, and imported to MZmine (version 2.53). We performed feature finding using the parameters recommended for GNPS Feature Based Molecular Networking (FBMN) (23). Particularly, mass detection was performed in centroid mode with a noise threshold of 1.0E3 for MS1 and 1.0E2 for MS2. Chromatograms were built with the ADAP Chromatogram Builder using a minimum group number of 5, group intensity threshold of 1.0E3, individual intensity threshold of 3.0E3, and m/z tolerance of 0.01. The local minimum search algorithm was used to deconvolute the chromatogram with a chromatographic threshold of 85%, search minimum of 0.01, minimum relative height of 50%, minimum absolute height of 3.0E3, minimum ratio of peak top / edge of 1.15, peak duration range between 0.01 and 10 min, m/z range of 0.02, and RT range of 0.15. Isotope peaks were removed with an m/z tolerance of 0.02, RT of 0.1 min, and maximum charge of 3. Processed peaks were aligned with an m/z tolerance of 0.02 and RT tolerance of 0.1 min, giving 75% weight to m/z tolerance and 25% weight to RT tolerance. This peak list was ordered, then filtered to have a minimum of 2 peaks in a row and 2 peaks within an isotope pattern. Only peaks that had an MS2 scan were retained. We exported the feature quantification table (.csv) and MS/MS spectral summary (.mgf) from MZmine, then imported them into GNPS and performed FBMN using release 28.2 with the default parameters (23, 55)

(vii) Metabolomics analyses: Shannon diversity (56) and Bray-Curtis dissimilarity (57) were calculated with the q2-diversity plugin in QIIME 2 2020.11 (50). Songbird's multinomial regression was performed on metabolite counts data using the formula "pths_status" (19). Mmvec's paired-omics function was performed to obtain conditional probabilities between the metabolomic counts data and shotgun metagenomics data (20). FBMN was performed within the GNPS environment with the default parameters in release 28.2 (23, 55). Graphml files were exported from the FBMN job into Cytoscape and colored by the proportion of summed ion intensity in PTHS individuals and their parents (58). The Chemical Explorer search within ReDU was utilized to infer associations between individual metabolites & other conditions (21). DEICODE's rclr function was used to perform the robust centered log transform on metabolite feature counts (59). Pearson correlation statistics, Kruskal-Wallis statistics, and Mann Whitney U statistics were calculated with SciPy (40). Plotting was performed with Seaborn and matplotlib using a Jupyter Notebook with Pandas.

(viii) Data / Code Availability: The raw metagenomic sequencing data is available in Qiita under the American Gut Project (study ID: 10317) and EBI accession ERP012803. The raw metabolomics data is available in MassIVE under ID MSV000083357. The FBMN job is available in GNPS with the following URL: https://gnps.ucsd.edu/ProteoSAFe/status.jsp?task=f2aa1c5a1f544b8fb67cf215d960f6a3. Jupyter notebooks containing the code to generate figures are available at https://github.com/ahdilmore/pths_notebooks.

Additional Information About Songbird and mmvec Analyses:

Songbird generates differentials, or log-fold changes of features relative to a specified field in the metadata. In our case Songbird generated a list of log-fold changes of metabolites relative to the ‘pths_status’ variable, so each differential represented how enrichment a given metabolite is in the PTHS group versus the unaffected parents group. Ranking the features is an important and useful way to examine the relative association of features (i.e. the top features are most associated with a given variable and the bottom variables are most associated with its opposite). Therefore, we examined the top metabolites to look at the metabolites most enriched in PTHS. For more information, see Songbird’s detailed README at https://github.com/biocore/songbird, the QIIME2 forum, or its associated publication (19).

mmvec’s paired-omics function generates a table of log conditional probabilities from a metabolite counts table and a microbial relative abundance table. Put more concretely, the final table is constructed with rows of metabolites and columns of microbes (or vice versa) and an individual cell represents the log probability that the microbe and metabolite that intersect at that cell occur together in the same samples. Again, ranking the log conditional probabilities is a useful way to interpret which metabolites are most associated with a given microbe or which microbes are most associated with a given metabolite. In other words, Songbird informs which microbes or metabolites are most associated with a particular metadata variable while mmvec informs which microbes and metabolites are most associated with each other. In our case, we were specifically interested in the microbe *C. bolteae*, so we used mmvec to identify metabolites associated with this microbe. For more information, see mmvec’s README at https://github.com/biocore/mmvec, the QIIME2 forum, or its associated publication (20).

Supplemental References:

28. McDonald D, Hyde E, Debelius JW, Morton JT, Gonzalez A, Ackermann G, Aksenov AA, Behsaz B, Brennan C, Chen Y, DeRight Goldasich L, Dorrestein PC, Dunn RR, Fahimipour AK, Gaffney J, Gilbert JA, Gogul G, Green JL, Hugenholtz P, Humphrey G, Huttenhower C, Jackson MA, Janssen S, Jeste DV, Jiang L, Kelley ST, Knights D, Kosciolek T, Ladau J, Leach J, Marotz C, Meleshko D, Melnik AV, Metcalf JL, Mohimani H, Montassier E, Navas-Molina J, Nguyen TT, Peddada S, Pevzner P, Pollard KS, Rahnavard G, Robbins-Pianka A, Sangwan N, Shorenstein J, Smarr L, Song SJ, Spector T, Swafford AD, Thackray VG, Thompson LR, Tripathi A, Vázquez-Baeza Y, Vrbanac A, Wischmeyer P, Wolfe E, Zhu Q, American Gut Consortium, Knight R. 2018. American Gut: an Open Platform for Citizen Science Microbiome Research. mSystems 3.

29. Amir A, McDonald D, Navas-Molina JA, Debelius J, Morton JT, Hyde E, Robbins-Pianka A, Knight R. 2017. Correcting for Microbial Blooms in Fecal Samples during Room-Temperature Shipping. mSystems 2.

30. Caporaso JG, Lauber CL, Walters WA, Berg-Lyons D, Huntley J, Fierer N, Owens SM, Betley J, Fraser L, Bauer M, Gormley N, Gilbert JA, Smith G, Knight R. 2012. Ultra-high-throughput microbial community analysis on the Illumina HiSeq and MiSeq platforms. ISME J 6:1621–1624.

31. Walters W, Hyde ER, Berg-Lyons D, Ackermann G, Humphrey G, Parada A, Gilbert JA, Jansson JK, Caporaso JG, Fuhrman JA, Apprill A, Knight R. 2016. Improved Bacterial 16S rRNA Gene (V4 and V4-5) and Fungal Internal Transcribed Spacer Marker Gene Primers for Microbial Community Surveys. mSystems 1.

32. Li H. 2018. Minimap2: pairwise alignment for nucleotide sequences. Bioinformatics

34:3094–3100.

33. Chen S, Zhou Y, Chen Y, Gu J. 2018. fastp: an ultra-fast all-in-one FASTQ preprocessor. Bioinformatics.

34. Gonzalez A, Navas-Molina JA, Kosciolek T, McDonald D, Vázquez-Baeza Y, Ackermann G, DeReus J, Janssen S, Swafford AD, Orchanian SB, Sanders JG, Shorenstein J, Holste H, Petrus S, Robbins-Pianka A, Brislawn CJ, Wang M, Rideout JR, Bolyen E, Dillon M, Caporaso JG, Dorrestein PC, Knight R. 2018. Qiita: rapid, web-enabled microbiome meta-analysis. Nat Methods 15:796–798.

35. Zhu Q, Mai U, Pfeiffer W, Janssen S, Asnicar F, Sanders JG, Belda-Ferre P, Al-Ghalith GA, Kopylova E, McDonald D, Kosciolek T, Yin JB, Huang S, Salam N, Jiao J-Y, Wu Z, Xu ZZ, Cantrell K, Yang Y, Sayyari E, Rabiee M, Morton JT, Podell S, Knights D, Li W-J, Huttenhower C, Segata N, Smarr L, Mirarab S, Knight R. 2019. Phylogenomics of 10,575 genomes reveals evolutionary proximity between domains Bacteria and Archaea. Nat Commun 10:5477.

36. Langmead B, Salzberg SL. 2012. Fast gapped-read alignment with Bowtie 2. Nat Methods 9:357–359.

37. Hillmann B, Al-Ghalith GA, Shields-Cutler RR, Zhu Q, Knight R, Knights D. 2020. SHOGUN: a modular, accurate and scalable framework for microbiome quantification. Bioinformatics 36:4088–4090.

38. Zhu Q, Huang S, Gonzalez A, McGrath I, McDonald D, Haiminen D, Armstrong G, Vázquez-Baeza Y, Yu J, Kuczynski J, Sepich-Poore GD, Swafford AD, Das P, Shaffer JP, Lejzerowicz F, Belda-Ferre P, Havulinna AS, Méric G, Niiranen T, Lahti L, Salomaa V, Kim HC, Jain M, Inouye M, Gilbert JA, Knight R. 2021. OGUs enable effective, phylogeny-

aware analysis of even shallow metagenome community structures. bioRxiv.

39. Aitchison J, Aitchison JW. 1986. The Statistical Analysis of Compositional Data. Springer Netherlands.

40. Virtanen P, Gommers R, Oliphant TE, Haberland M, Reddy T, Cournapeau D, Burovski E, Peterson P, Weckesser W, Bright J, van der Walt SJ, Brett M, Wilson J, Millman KJ, Mayorov N, Nelson ARJ, Jones E, Kern R, Larson E, Carey CJ, Polat İ, Feng Y, Moore EW, VanderPlas J, Laxalde D, Perktold J, Cimrman R, Henriksen I, Quintero EA, Harris CR, Archibald AM, Ribeiro AH, Pedregosa F, van Mulbregt P, SciPy 1.0 Contributors. 2020. SciPy 1.0: fundamental algorithms for scientific computing in Python. Nat Methods 17:261–272.

41. Waskom M. 2021. seaborn: statistical data visualization. J Open Source Softw 6:3021.

42. Hunter. 2007. Matplotlib: A 2D Graphics Environment 9:90–95.

43. Amir A, McDonald D, Navas-Molina JA, Kopylova E, Morton JT, Zech Xu Z, Kightley EP, Thompson LR, Hyde ER, Gonzalez A, Knight R. 2017. Deblur Rapidly Resolves Single-Nucleotide Community Sequence Patterns. mSystems 2.

44. McDonald D, Kaehler B, Gonzalez A, DeReus J, Ackermann G, Marotz C, Huttley G, Knight R. 2019. redbiom: a Rapid Sample Discovery and Feature Characterization System. mSystems.

45. McDonald D, Clemente JC, Kuczynski J, Rideout JR, Stombaugh J, Wendel D, Wilke A, Huse S, Hufnagle J, Meyer F, Knight R, Caporaso JG. 2012. The Biological Observation Matrix (BIOM) format or: how I learned to stop worrying and love the ome-ome. Gigascience 1:7.

46. DeSantis TZ, Hugenholtz P, Larsen N, Rojas M, Brodie EL, Keller K, Huber T, Dalevi D, Hu P, Andersen GL. 2006. Greengenes, a chimera-checked 16S rRNA gene database and workbench compatible with ARB. Appl Environ Microbiol 72:5069–5072.

47. McDonald D, Price MN, Goodrich J, Nawrocki EP, DeSantis TZ, Probst A, Andersen GL, Knight R, Hugenholtz P. 2012. An improved Greengenes taxonomy with explicit ranks for ecological and evolutionary analyses of bacteria and archaea. ISME J 6:610–618.

48. Janssen S, McDonald D, Gonzalez A, Navas-Molina JA, Jiang L, Xu ZZ, Winker K, Kado DM, Orwoll E, Manary M, Mirarab S, Knight R. 2018. Phylogenetic Placement of Exact Amplicon Sequences Improves Associations with Clinical Information. mSystems 3.

49. Faith DP. 1992. Conservation evaluation and phylogenetic diversity. Biol Conserv 61:1–10.

50. Bolyen E, Rideout JR, Dillon MR, Bokulich NA, Abnet CC, Al-Ghalith GA, Alexander H, Alm EJ, Arumugam M, Asnicar F, Bai Y, Bisanz JE, Bittinger K, Brejnrod A, Brislawn CJ, Brown CT, Callahan BJ, Caraballo-Rodríguez AM, Chase J, Cope EK, Da Silva R, Diener C, Dorrestein PC, Douglas GM, Durall DM, Duvallet C, Edwardson CF, Ernst M, Estaki M, Fouquier J, Gauglitz JM, Gibbons SM, Gibson DL, Gonzalez A, Gorlick K, Guo J, Hillmann B, Holmes S, Holste H, Huttenhower C, Huttley GA, Janssen S, Jarmusch AK, Jiang L, Kaehler BD, Kang KB, Keefe CR, Keim P, Kelley ST, Knights D, Koester I, Kosciolek T, Kreps J, Langille MGI, Lee J, Ley R, Liu Y-X, Loftfield E, Lozupone C, Maher M, Marotz C, Martin BD, McDonald D, McIver LJ, Melnik AV, Metcalf JL, Morgan SC, Morton JT, Naimey AT, Navas-Molina JA, Nothias LF, Orchanian SB, Pearson T, Peoples SL, Petras D, Preuss ML, Pruesse E, Rasmussen LB, Rivers A, Robeson MS 2nd, Rosenthal P, Segata N, Shaffer M, Shiffer A, Sinha R, Song SJ, Spear JR, Swafford AD, Thompson LR, Torres PJ, Trinh P, Tripathi A, Turnbaugh PJ, Ul-Hasan S, van der Hooft JJJ, Vargas F, Vázquez-Baeza Y, Vogtmann E, von Hippel M, Walters W, Wan Y, Wang M, Warren J, Weber KC,

Williamson CHD, Willis AD, Xu ZZ, Zaneveld JR, Zhang Y, Zhu Q, Knight R, Caporaso JG. 2019. Reproducible, interactive, scalable and extensible microbiome data science using QIIME 2. Nat Biotechnol 37:852–857.

51. McKinney W. 2010. Data Structures for Statistical Computing in PythonProceedings of the 9th Python in Science Conference. SciPy.

52. Reback J, jbrockmendel, McKinney W, Van den Bossche J, Augspurger T, Cloud P, Hawkins S, gfyoung, Sinhrks, Roeschke M, Klein A, Petersen T, Tratner J, She C, Ayd W, Hoefler P, Naveh S, Garcia M, Schendel J, Hayden A, Saxton D, Gorelli ME, Shadrach R, Jancauskas V, McMaster A, Li F, Battiston P, Seabold S, attack, Dong K. 2021. pandas-dev/pandas: Pandas 1.3.0.

53. Fang X, Vázquez-Baeza Y, Elijah E, Vargas F, Ackermann G, Humphrey G, Lau R, Weldon KC, Sanders JG, Panitchpakdi M, Carpenter C, Jarmusch AK, Neill J, Miralles A, Dulai P, Singh S, Tsai M, Swafford AD, Smarr L, Boyle DL, Palsson BO, Chang JT, Dorrestein PC, Sandborn WJ, Knight R, Boland BS. 2021. Gastrointestinal Surgery for Inflammatory Bowel Disease Persistently Lowers Microbiome and Metabolome Diversity. Inflamm Bowel Dis 27:603–616.

54. Aron AT, Gentry EC, McPhail KL, Nothias L-F, Nothias-Esposito M, Bouslimani A, Petras D, Gauglitz JM, Sikora N, Vargas F, van der Hooft JJJ, Ernst M, Kang KB, Aceves CM, Caraballo-Rodríguez AM, Koester I, Weldon KC, Bertrand S, Roullier C, Sun K, Tehan RM, Boya P CA, Christian MH, Gutiérrez M, Ulloa AM, Tejeda Mora JA, Mojica-Flores R, Lakey-Beitia J, Vásquez-Chaves V, Zhang Y, Calderón AI, Tayler N, Keyzers RA, Tugizimana F, Ndlovu N, Aksenov AA, Jarmusch AK, Schmid R, Truman AW, Bandeira N, Wang M, Dorrestein PC. 2020. Reproducible molecular networking of untargeted mass spectrometry data using GNPS. Nat Protoc 15:1954–1991.

55. Wang M, Carver JJ, Phelan VV, Sanchez LM, Garg N, Peng Y, Nguyen DD, Watrous J, Kapono CA, Luzzatto-Knaan T, Porto C, Bouslimani A, Melnik AV, Meehan MJ, Liu W-T, Crüsemann M, Boudreau PD, Esquenazi E, Sandoval-Calderón M, Kersten RD, Pace LA, Quinn RA, Duncan KR, Hsu C-C, Floros DJ, Gavilan RG, Kleigrewe K, Northen T, Dutton RJ, Parrot D, Carlson EE, Aigle B, Michelsen CF, Jelsbak L, Sohlenkamp C, Pevzner P, Edlund A, McLean J, Piel J, Murphy BT, Gerwick L, Liaw C-C, Yang Y-L, Humpf H-U, Maansson M, Keyzers RA, Sims AC, Johnson AR, Sidebottom AM, Sedio BE, Klitgaard A, Larson CB, P CAB, Torres-Mendoza D, Gonzalez DJ, Silva DB, Marques LM, Demarque DP, Pociute E, O'Neill EC, Briand E, Helfrich EJN, Granatosky EA, Glukhov E, Ryffel F, Houson H, Mohimani H, Kharbush JJ, Zeng Y, Vorholt JA, Kurita KL, Charusanti P, McPhail KL, Nielsen KF, Vuong L, Elfeki M, Traxler MF, Engene N, Koyama N, Vining OB, Baric R, Silva RR, Mascuch SJ, Tomasi S, Jenkins S, Macherla V, Hoffman T, Agarwal V, Williams PG, Dai J, Neupane R, Gurr J, Rodríguez AMC, Lamsa A, Zhang C, Dorrestein K, Duggan BM, Almaliti J, Allard P-M, Phapale P, Nothias L-F, Alexandrov T, Litaudon M, Wolfender J-L, Kyle JE, Metz TO, Peryea T, Nguyen D-T, VanLeer D, Shinn P, Jadhav A, Müller R, Waters KM, Shi W, Liu X, Zhang L, Knight R, Jensen PR, Palsson BO, Pogliano K, Linington RG, Gutiérrez M, Lopes NP, Gerwick WH, Moore BS, Dorrestein PC, Bandeira N. 2016. Sharing and community curation of mass spectrometry data with Global Natural Products Social Molecular Networking. Nat Biotechnol 34:828–837.

56. Shannon CE. 1948. A Mathematical Theory of Communication. Bell System Technical Journal.

57. Bray JR, Roger Bray J, Curtis JT. 1957. An Ordination of the Upland Forest Communities of Southern Wisconsin. Ecological Monographs.

58. Shannon P, Markiel A, Ozier O, Baliga NS, Wang JT, Ramage D, Amin N, Schwikowski B,

Ideker T. 2003. Cytoscape: a software environment for integrated models of biomolecular interaction networks. Genome Res 13:2498–2504.

59. Martino C, Morton JT, Marotz CA, Thompson LR, Tripathi A, Knight R, Zengler K. 2019. A Novel Sparse Compositional Technique Reveals Microbial Perturbations. mSystems 4.

60. Lozupone C, Knight R. 2005. UniFrac: a new phylogenetic method for comparing microbial communities. Appl Environ Microbiol 71:8228–8235.
