# Supplementary material for: The Fecal Microbiome and Metabolome of Pitt Hopkins Syndrome, a Severe Autism Spectrum Disorder
Source: mSystems. 2021 Nov 30;6(6):e01006-21. doi: 10.1128/mSystems.01006-21 (PMC8631314; doi:10.1128/mSystems.01006-21)
Supplement: TABLE S1 [file msystems.01006-21-st001.pdf]

Feature ID	m/z	RT	Annotation & Level	Identified from?	Log ratio / Conditional Probability
**1914**	300.29010	4.50	N/A - Level 4	Songbird	4.291592
**1948**	311.29490	6.97	Spectral Match to 1-Stearoyl-2- hydroxy-sn-glycero-3-phosphocholine from NIST14 - Level 3	Songbird	3.489643
**2296**	229.15480	0.25	N/A - Level 4	Songbird	3.346251
**2670**	159.14940	0.38	N/A - Level 4	Songbird	2.822391
**1554**	282.27940	4.53	N/A - Level 4	Songbird	2.776787
**2000**	304.30020	4.85	N/A - Level 4	Songbird	2.408696
**1263**	288.29000	4.32	N/A - Level 4	Songbird	2.364576
**2857**	349.27190	7.16	N/A - Level 4	Songbird	2.332370
**2037**	407.27930	3.30	Spectral match to (R)-4-((1R,3S,5S,7R,8S,9S,10S,12S,13R,14S,17R)-1,3,7,12-tetrahydroxy-10,13-dimethylhexadecahydro-1H-cyclopenta[a]phenanthren-17-yl)pentanoic acid - Level 3	Songbird	2.188757
**2697**	593.47760	7.56	N/A - Level 4	Songbird	2.037039

**4291**	431.27690	3.57	Spectral match to (R)-4-((3R,5S,7R, 8R,9S,10S,12S,13R,14S,17R)-3,7,12-trihydroxy-10,13-dimethylhexadecahydro-1H-cyclopenta[a]phenanthren-17-yl)pentanoic acid" - Level 3	Songbird	1.965308
**1547**	302.30570	4.70	Spectral match to D-erythro-Dihydrosphingosine764-22-7 - Level 2	Songbird	1.916654
**885**	161.02960	0.24	N/A - Level 4	Songbird	1.789181
**1456**	393.29780	7.21	N/A - Level 4	Songbird	1.782732
**2676**	577.48180	7.58	N/A - Level 4	Songbird	1.754848
**890**	283.26340	5.56	N/A - Level 4	mmvec	4.216325
**8**	338.34230	7.91	N/A - Level 4	mmvec	3.527352
**2647**	601.53980	5.55	N/A - Level 4	mmvec	3.444107
**1526**	385.34720	9.08	Spectral Match to (+)-4-Cholesten- 3-one from NIST14 - Level 3	mmvec	3.348641
**898**	265.25290	5.56	N/A - Level 4	mmvec	3.230710
**1528**	403.28240	6.68	N/A - Level 4	mmvec	3.229489
**1277**	323.25600	5.56	N/A - Level 4	mmvec	3.212855

**900**	321.24040	5.77	N/A - Level 4	mmvec	3.051383
**1394**	429.37340	9.41	N/A - Level 4	mmvec	2.991190
**1070**	299.25840	5.77	N/A - Level 4	mmvec	2.908168
**1535**	321.27910	5.97	N/A - Level 4	mmvec	2.625159
**1279**	171.09950	2.28	N/A - Level 4	mmvec	2.547605
**1826**	301.27340	5.55	N/A - Level 4	mmvec	2.545458
**914**	305.24560	7.10	N/A - Level 4	mmvec	2.509711

1
